# Branched-chain amino acids and Alzheimer’s disease: a Mendelian randomization analysis

**DOI:** 10.1038/s41598-017-12931-1

**Published:** 2017-10-19

**Authors:** Susanna C. Larsson, Hugh S. Markus

**Affiliations:** 1 Unit of Nutritional Epidemiology, Institute of Environmental Medicine, Karolinska Institutet, Stockholm, Sweden; 2 Stroke Research Group, Department of Clinical Neurosciences, University of Cambridge, Cambridge, United Kingdom

## Abstract

We conducted a two-sample Mendelian randomization study to test the hypothesis that raised plasma levels of the branched-chain amino acids isoleucine, leucine, and valine are associated with Alzheimer’s disease (AD). From a genome-wide association study of 16,596 individuals of European ancestry, we obtained summary statistics for four independent single nucleotide polymorphisms (SNPs) associated with isoleucine levels and one SNP associated with both leucine and valine levels at genome-wide significance. Summary statistics of the associations of the five SNPs with AD were obtained from the International Genomics of Alzheimer’s Project (17,008 AD cases and 37,154 controls). Based on four SNPs, the odds ratio of AD per genetically predicted one standard deviation higher isoleucine levels was 1.35 (95% CI, 1.08–1.69; *p* = 0.007). The leucine- and valine-raising allele was not associated with AD (*p* = 0.46). These data suggest that a genetic predisposition to raised plasma isoleucine levels is positively associated with AD.

## Introduction

Alzheimer’s disease (AD) is a neurodegenerative disease characterized by synaptic loss and neuronal cell death, primarily in hippocampus, and manifests as progressive memory loss and cognitive decline. The pathological hallmarks of AD are amyloid plaques containing deposits of amyloid-β (Aβ) peptides, and neurofibrillary tangles containing hyperphosphorylated tau protein^1^. The neuronal damage that leads to AD may in part be caused by toxic effect of Aβ peptides or alterations in Aβ processing^1^. Thus, modifiable environmental factors or medical therapies that modify the accumulation of amyloid plaques or hippocampal neurogenesis may influence AD development.

The neurotransmitter serotonin is a candidate for reducing amyloid plaque load^2–5^ and for enhancing neurogenesis^2,6–8^. It has been observed that AD patients have decreased serotonin receptor levels^9–12^ and that activation of serotonin receptors decreases Aβ production^5,13^ and brain Aβ levels^2–5^ and increases neuronal survival^2^. There are also data showing that selective serotonin reuptake inhibitors, a class of antidepressants that increase serotonin levels, reduce brain Aβ levels^3,4^ and stimulate neurogenesis in hippocampus^6–8^.

**Figure 1:**
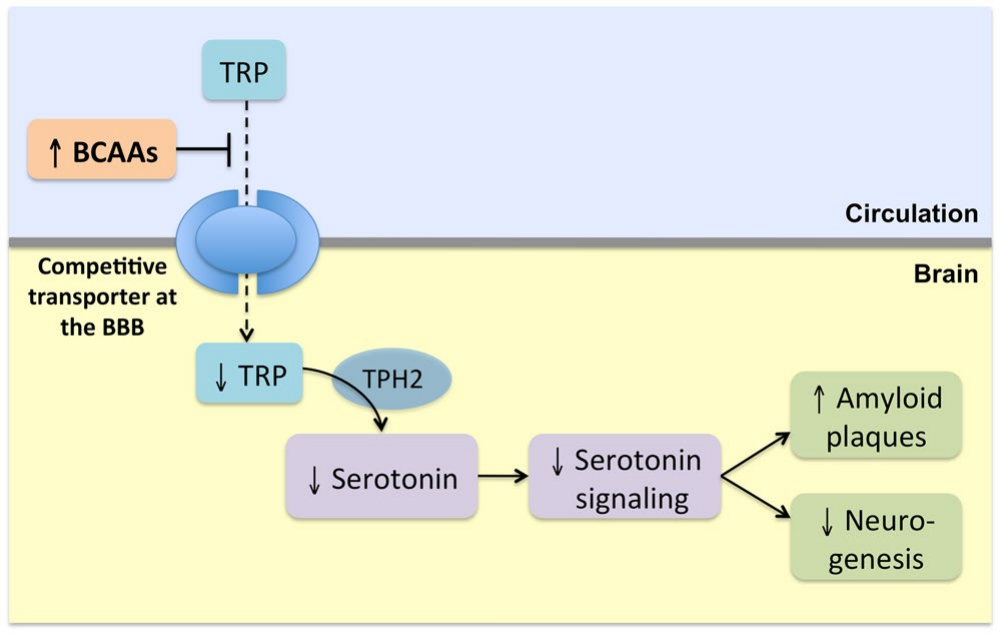
Hypothetical effects of raised circulating levels of branched-chain amino acids. Tryptophan (TRP) is converted to serotonin in neurons by tryptophan hydroxylase 2 (TPH2), the rate-limiting enzyme in serotonin synthesis. Because this enzyme is normally unsaturated with TRP, brain serotonin levels are controlled by TRP levels. Branched-chain amino acids (BCAAs) reduce the uptake of TRP into the brain by competing for transport at the blood-brain barrier (BBB). Lifelong elevated BCAA levels conferred by genetic predisposition to higher plasma BCAA levels may therefore have long-term effects on serotonin signaling and downstream pathways that may influence the risk of Alzheimer’s disease.

The branched-chain amino acids (BCAAs) isoleucine, leucine, and valine are essential amino acids that are obtained from the diet through protein-containing foods. Uptake of BCAAs into the brain occurs at the blood-brain barrier through a competitive transport carrier that they share with other large neutral amino acids, including tryptophan, the precursor of serotonin^14–16^. Long-term raised circulating BCAA levels could conceivably lead to decreased brain tryptophan levels as well as reduced neuronal serotonin synthesis and serotonergic signaling^15–17^ (Fig. 1). This may consequently increase AD risk but this hypothesis has, to our knowledge, not yet been tested.

Mendelian randomization (MR) is a genetic epidemiologic approach that utilizes genetic variants associated with the modifiable exposure (e.g., plasma BCAA levels) as proxies for the exposure to evaluate whether the exposure is causally associated with the outcome (e.g., AD). This method reduces some of the crucial limitations of observational studies, including confounding and reverse causation bias. A recent genome-wide association study (GWAS) identified several genetic variants that are associated with plasma BCAA levels^18^. The aim of the present study was to use MR to test the hypothesis that plasma BCAA levels are positively associated with AD.

## Methods

### Selection of genetic variants and assessment of pleiotropy

A GWAS of 16,596 individuals of European ancestry identified five independent genomic regions with single-nucleotide polymorphisms (SNPs) associated with plasma BCAA levels at genome wide significance level (*p* < 5 × 10^−8^)^18^. We selected the lead SNP from each of these genomic regions. Five independent SNPs (R^2^ < 0.03) were associated with isoleucine levels. These SNPs were located near *PPM1K* (rs7678928), *DDX19A* (rs75950518), *TRMT61A* (rs58101275), *CBLN1* (rs1420601), and *GCKR* (rs1260326). Only one SNP (rs1440581), near the *PPM1K* gene, was associated with leucine and valine levels at genome-wide significance. To investigate whether the BCAA-associated SNPs had pleiotropic associations with other phenotypes, we searched PhenoScanner, which is a publicly available GWAS database^19^.

### Association with AD

Summary statistics (beta coefficients and standard errors) for the associations of the BCAA-related SNPs with AD were obtained from the International Genomics of Alzheimer’s Project (IGAP)^20^. IGAP is a 2-stage study based upon GWAS on individuals of European descent^20^. We used data from the first stage of IGAP, which genotyped and imputed data on 7,055,881 SNPs to meta-analyze 4 GWAS datasets [Alzheimer Disease Genetics Consortium (ADGC), the Cohorts for Heart and Aging Research in Genomic Epidemiology consortium (CHARGE), the European Alzheimer’s disease Initiative (EADI), and the Genetic and Environmental Risk in AD consortium (GERAD)], including a total of 17,008 cases of AD and 37,154 controls. All studies included in IGAP had been approved by a relevant Institutional Review Board. Informed consent was obtained from participants themselves or, for those with considerable cognitive impairment, from a caregiver, legal guardian, or other proxy.

### Statistical analysis

An instrumental variable was constructed for each BCAA-associated SNP by dividing the SNP’s effect (beta coefficient) on AD by the corresponding effect on BCAA levels. The instrumental variable estimates for isoleucine were combined using a fixed-effect inverse-variance weighted meta-analysis^21^. To explore and adjust for pleiotropy, sensitivity analyses were performed using the weighted median and MR-Egger regression methods^21^. Results are reported as odds ratios (OR) with their 95% confidence intervals (CI) of AD per genetically predicted 1 standard deviation (SD) increase in plasma isoleucine, leucine, and valine levels. All statistical tests were two-sided. We applied a Bonferroni-corrected significance threshold for analyses of five BCAA-raising SNPs (*p* = 0.05/5 = 0.01). Tests were otherwise considered statistically significant at *p* < 0.05. The statistical analyses were conducted using Stata, version 14.2 (StataCorp, College Station, Texas, USA) and R (R Foundation), version 3.3.3.

### Availability of data and materials

All data generated or analyzed during this study are included in this published article and its supplementary information file.

## Results

**Figure 2:**
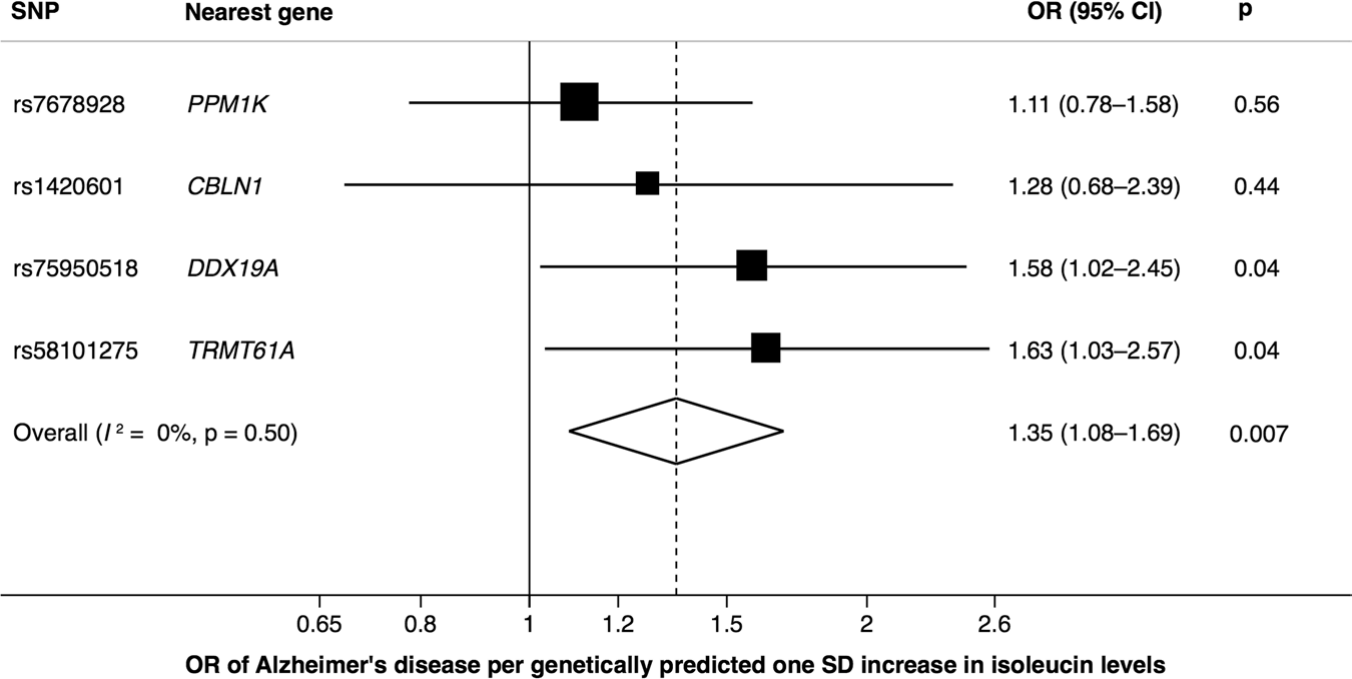
Association between genetically predicted isoleucine levels and Alzheimer’s disease. Diamond represents the overall odds ratio (OR) with its 95% confidence interval (CI) of Alzheimer’s disease. SD: standard deviation.

One SNP (rs1260326 near the *GCKR* gene) had strong pleiotropic associations with numerous phenotypes (Table S1), and was excluded from the analysis. The remaining SNPs, including four SNPs associated with isoleucine and one SNP associated with leucine and valine, were used as instrumental variables in the MR analyses. All four isoleucine-raising alleles were positively associated with AD but none of the associations was significant at the Bonferroni-corrected significance threshold (Fig. 2; Table S2). The overall OR of AD per genetically predicted 1 SD increase in isoleucine levels, based on four SNPs, was 1.35 (95% CI, 1.08–1.69; *p* = 0.007), without heterogeneity among estimates from different SNPs (*I* ^2^ = 0%, *p* = 0.50) (Fig. 2). The association was consistent in a sensitivity analysis using the weighted median method (OR = 1.35; 95% CI 1.03–1.77; *p* = 0.03). The MR-Egger analysis showed no evidence of pleiotropy (intercept = −0.029, *p* = 0.71) but the causal estimate from this analysis was very imprecise and the CI included the null (OR, 1.87; 95% CI, 0.33–10.58). The leucine- and valine-raising allele was not significantly associated with AD (*p* = 0.46) (Table S2). The ORs per genetically predicted 1 SD increase in genetically predicted leucine and valine levels were 1.16 (95% CI, 0.78–1.72) and 1.13 (95% CI, 0.82–1.57), respectively.

## Discussion

This MR analysis showed that a genetic predisposition to higher plasma isoleucine levels was positively associated with AD. This finding suggests that lifelong raised isoleucine levels may increase the risk of AD. Genetically predicted higher levels of leucine and valine, based on a single genetic variant, were not associated with AD.

It is well known that an increase in circulating BCAA levels leads to decreased brain levels of tryptophan and its conversion to serotonin (Fig. 1)^15,16,22^. This effect reflects the competitive nature of the blood-brain barrier large neutral amino acid transporter, which is almost fully saturated at normal plasma levels of these amino acids^22^. Hence, the observed association of genetically raised isoleucine levels with AD may be mediated by decreased brain serotonin levels and diminished serotonin signaling, potentially leading to higher amyloid plaque load^2–5^ and decreased neuronal survival^2^ and adult hippocampal neurogenesis^6–8^. Experimental data have shown that dietary BCAA supplementation leads to lowered hippocampal levels of nerve growth factor, which is involved in growth and survival of cholinergic neurons^23^. High BCAA levels also result in reduced brain uptake of tyrosine and its conversion to the catecholamine neurotransmitters dopamine and norepinephrine^15,22^, but their role in AD is uncertain.

A strength of the MR approach is that potential confounding from other modifiable risk factors were reduced by the use of genetic variants as proxies for BCAA exposure. However, we cannot rule out the possibility that the BCAA-associated genetic variants may affect the risk of AD through pathways other than through BCAA levels. For this reason, we excluded the pleiotropic variant near the *GCKR* gene. The genetic risk scores for isoleucine, leucine and valine were specifically associated with the three BCAAs and not with any of 172 other metabolites measured^18^, suggesting that pleiotropic effects with other metabolites unlikely explain our findings. We also found no evidence of pleiotropy in sensitivity analyses using the weighted median and MR-Egger methods. However, the MR-Egger method had low statistical power and could not reliably detect pleiotropy. The genetic variants were weakly associated, at nominal statistical significance, with some diseases and disorders, most notably psychiatric and mood disorders that are related to impaired serotonin signaling, such as schizophrenia, bipolar disorders, and major depressive disorders. This observation reinforces the hypothesis that genetically raised BCAA levels might increase AD risk by modifying the serotonergic system.

A limitation of this study is that our genetic instrument for leucine and valine comprised only one genetic variant, leading to low statistical power to detect an association. Although the odds ratio estimates for the leucine- and valine-raising allele was in the same direction as for isoleucine, the precision was low and the associations were not significant. Likewise, none of the individual SNPs associated with isoleucine levels was significantly associated with AD at the Bonferroni-corrected significance level.

In conclusion, using data from a large genetic consortium on AD, we found that a genetic predisposition to raised plasma isoleucine levels was associated with AD. The role of BCAAs in the development of AD merits further investigation.

## Electronic supplementary material

Supplementary Material
