# Supplementary material for: Branched-chain amino acids and Alzheimer’s disease: a Mendelian randomization analysis
Source: Sci Rep. 2017 Oct 19;7:13604. doi: 10.1038/s41598-017-12931-1 (PMC5648806; doi:10.1038/s41598-017-12931-1)
Supplement: Supplementary file 1 — Supplementary Material [file 41598_2017_12931_MOESM1_ESM.pdf]

Supplementary Material

Branched-chain amino acids and Alzheimer's disease: a Mendelian randomization analysis

Authors: Susanna C. Larsson & Hugh S. Markus

Supplementary tables

**Table S1**	Associations of the BCAA-associated single nucleotide polymorphisms with other phenotypes at $p < 0.05$ in published genome-wide association studies	2
**Table S2**	Characteristics of the BCAA-associated single nucleotide polymorphisms and their associations with Alzheimer's disease	3

Table S1. Associations of the BCAA-associated single nucleotide polymorphisms with other phenotypes at $p < 0.05$ in published genome-wide association studies

BCAA	SNP	Nearest gene	Phenotype	*p* value	PMID
Isoleucine	rs7678928	*PPM1K*	Serum urate	2.4×10^{-4}	23263486
			Irritable bowel syndrome	0.002	18587394
			Geographic atrophy	0.005	23455636
			Bipolar disorder	0.007	22182935
			Age-related macular degeneration	0.013	23455636
			Crohn's disease	0.016	23128233
			Obesity class 1	0.022	23563607
			2 hour glucose	0.044	20081857
Isoleucine	rs1260326*	*GCKR*	Triglycerides	2.0×10^{-239}	24097068
			Serum urate	1.3×10^{-44}	23263486
			C-reactive protein	3.8×10^{-43}	23263486
			Fasting glucose	2.2×10^{-41}	22885924
			HDL cholesterol	6.3×10^{-36}	19936222
			Coagulation factor VII	1.7×10^{-28}	21676895
			Total cholesterol	7.0×10^{-27}	20686565
			Fasting insulin	2.7×10^{-22}	22885924
			Chronic kidney disease	3.0×10^{-14}	20383146
			Gout	2.0×10^{-12}	25646370
			Serum creatinine	3.4×10^{-12}	20383146
			Apolipoprotein C	8.7×10^{-12}	19060906
			Height	1.4×10^{-11}	25282103
			Type 2 diabetes	3.7×10^{-9}	26551672
			Age at menopause	1.0×10^{-9}	26414677
			Plasma palmitoleic acid	3.8×10^{-9}	23362303
			Alcohol consumption	2.3×10^{-8}	28485404
			Coffee consumption	7.0×10^{-8}	25288136
			Leptin	3.6×10^{-7}	26833098
			HOMA insulin resistance	9.2×10^{-7}	20081858
			Apolipoprotein A	9.9×10^{-7}	19802338
			Apolipoprotein B	1.6×10^{-6}	19802338
			Crohn's disease	2.3×10^{-6}	18587394
			Body mass index	4.6×10^{-5}	25673413
			LDL cholesterol	2.3×10^{-4}	20686565
Isoleucine	rs75950518	*DDX19A*	Lumber spine bone mineral density	0.019	26367794
			Femoral neck mineral density	0.021	26367794
Isoleucine	rs58101275	*TRMT61A*	Schizophrenia	0.004	25056061
			Femoral neck mineral density	0.004	26367794
			Crohn's disease	0.015	26192919
			Neuroticism	0.026	27089181
Isoleucine	rs1420601	*CBLN1*	Major depression disorder	0.003	26176920
			Schizophrenia	0.007	25056061
			Myocardial infarction	0.037	26343387
Leucine/valine	rs1440581	*PPM1K*	Metabolite levels	1.0×10^{-16}	24816252
			Serum Fischers ratio	2.0×10^{-16}	22286219
			Serum alanine/valine and valine	3.8×10^{-15}	22286219

*Only the major phenotypes are listed because of the large number of pleiotropic associations. This SNP was excluded from the analysis. BCAA: branched-chain amino acid; SNP: single nucleotide polymorphism.

Table S2. Characteristics of the BCAA-associated single nucleotide polymorphisms and their associations with Alzheimer's disease

BCAA	SNP	Chr	Nearest gene	EA/ NEA	EAF	Beta (SE) of BCAA levels per EA	*p* value for BCAA	OR (95% CI) of AD per allele	*p* value for AD
Isoleucine	rs7678928	4	*PPM1K*	T/C	46%	0.090 (0.0125)	5.6×10^{-19}	1.010 (0.978–1.042)	0.56
Isoleucine	rs75950518	16	*DDX19A*	C/T	89%	0.107 (0.0190)	2.1×10^{-08}	1.050 (1.002–1.101)	0.04
Isoleucine	rs58101275	14	*TRMT61A*	G/A	79%	0.085 (0.0153)	2.8×10^{-08}	1.042 (1.003–1.084)	0.04
Isoleucine	rs1420601	16	*CBLN1*	C/T	40%	0.069 (0.0125)	3.7×10^{-08}	1.017 (0.974–1.062)	0.44
Leucine	rs1440581	4	*PPM1K*	C/T	53%	0.081 (0.0132)	3.9×10^{-25}	1.012 (0.980–1.045)	0.46
Valine	rs1440581	4	*PPM1K*	C/T	53%	0.098 (0.0133)	4.4×10^{-24}	1.012 (0.980–1.045)	0.46

AD: Alzheimer's disease; BCAA: branched-chain amino acid; Chr: chromosome; CI: confidence interval; EA: effect allele; EAF: effect allele frequency; NEA: non-effect allele; OR: odds ratio; SE: standard error; SNP: single-nucleotide polymorphism.
